# Supporting Decarbonization of Health Systems—A Review of International Policy and Practice on Health Care and Climate Change

**DOI:** 10.1007/s40572-024-00434-x

**Published:** 2024-02-15

**Authors:** Emily Hough, Arielle Cohen Tanugi-Carresse

**Affiliations:** 1 NHS Mid and South Essex Integrated Care Board, Essex, UK; 2 Brown University, Providence, RI USA; 3 ERUDITE, Université Paris-Est Créteil, Créteil, France

**Keywords:** Health system, Climate change, Decarbonization, International policy, Mitigation, Adaptation

## Abstract

**Purpose of Review:**

Healthcare is a significant contributor of carbon emissions, which contribute to climate change. There has been an increased focus on the role healthcare should play in reducing emissions in recent years. This review, completed in September and October 2022, explored national commitments among 73 countries to reduce emissions from healthcare and the policies and delivery plans that exist to support their implementation.

**Recent Findings:**

Whilst some countries such as Norway, Columbia, and Australia are working to understand current emissions and develop plans to reduce them, few have published delivery plans for meeting national targets. Broader policies and reports published to date provide a clear set of actions that healthcare can take to reduce emissions. However, more research, innovation, and service redesign will be needed to close the gap to net zero healthcare.

**Summary:**

Some health systems are already taking action to reduce their emissions. However, national incentives, including standardized metrics and reporting, can help drive broader action and pace of delivery.

## Introduction

Climate change is impacting populations across the world, with some describing it as the “greatest threat facing humanity” [1]. As health faces this threat, it has an opportunity to drive transformation of care and health systems to reduce its own emissions and ensure capacity to meet future demand. Climate change can harm human health and wellbeing in many ways across a variety of time scales, leading to illness and death. For example, extreme heat, drought and poor air quality can lead to respiratory complications and exacerbations in existing health conditions; while flooding and storms can increase spread of infectious diseases [2]. Climate change is also impacting mental health, causing grief and trauma in those who have experienced climate-related disasters, losing possessions and loved ones, and increased anxiety and depression related to climate-anxiety [3]. 

Climate-related events can also cause significant damage to health systems and disrupt immediate and long-term provision of care. Wildfires and floods can impact immediate access for patients and goods, while storms and hurricanes can close hospitals or cause power cuts that stop people accessing essential treatments. Longer term supply of essential goods can also be affected, as demonstrated when Hurricane Maria damaged a key saline manufacturing plant that supplied the US [4]. The impacts of climate change do not fall equally across populations; people of color, those with low income, older adults, people with disabilities, and children can be impacted more than others [5•]. Climate change is both a health crisis and an equity crisis.

Healthcare is often the first line of defense in supporting individuals and communities as they respond to and recover from the impacts of climate change. However, the sector is a high carbon emitter, with the US healthcare system is one of the world’s worst offenders, contributing 8.5% of the nation’s carbon emissions [6•]. By comparison, in the US 28% of emissions comes from transport and 25% from electricity generation [7]. Health organizations have both the opportunity and obligation to reduce emissions that come with the industry’s size, its large carbon footprint, and its mission to improve health.

Dedicated discussions on the connections between climate change and health are now a regular feature at international convenings such as the United Nations Climate Change Conference of the Parties (COPs) and the World Economic Forum (WEF). COP26 (2021) and COP27 (2022) both featured sessions on climate change and health, allowing an increased focus on both the mitigation and adaptation agendas. COP28 in Dubai, UAE, was the first UN climate conference to feature an official Health Day, with 147 countries officially signing the COP28 Declaration on Climate and Health, over 200 climate-health events, the highest number of health professionals and ministers of health, and over $1 billion funding commitment to climate and health [8]. The impacts of climate and health was also part of key panel discussions at the 2023 WEF and the need for investment to support action is being recognized by global foundations such as Wellcome, who have set Climate and Health as one of their three priorities [9, 10].

**Fig. 1:**
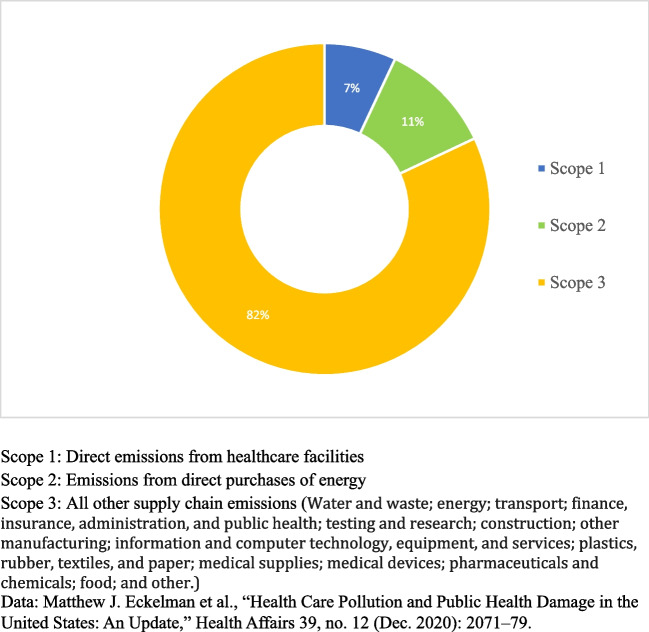
Greenhouse gas emissions from the US Health Sector, by Scope (2018)

Health systems across the world are starting to take action to address the impacts of climate change on health. This includes action to decarbonize (reduce or eliminate carbon dioxide emissions) across their own systems, and to adapt and prepare for the changing demand for care driven by climate change. The greatest challenge in this will be in decarbonizing the supply chain, which is the largest contributor to healthcare’s carbon footprint (see Fig. 1). The need to reduce overall emissions and shift to clean, renewable energy and increase energy efficiency is not unique to healthcare and there is much that health can learn from other sectors in reducing emissions from buildings, transport, and energy. In the US, around 11% of emissions come from directly purchased energy (see Fig. 1), with some estimates suggesting energy across the supply chain accounts for as much as 35% of healthcare emissions [11, 12]. In addition, hospitals tend to be inefficient and use significantly more energy than other buildings. Hospital buildings use an average of 250 kilo British Thermal Units (kBTU) per square foot, nearly three times that of typical commercial buildings [13].

This review sought to identify international commitments and policies relating to climate smart, low carbon sustainable health systems with a view to identifying actions and enablers that could be adapted to US policy contexts. The review highlights how different nations are approaching adaptation and mitigation of climate change impacts on health and outlines specific opportunities for reducing emissions from healthcare.

## Methods

A review of literature relating to international policy and practice on health system decarbonization was completed between September and October 2022 using online search engines (Google and Ecosia) and an agreed set of search terms relating to climate change and health. Search terms used to inform the review included the following: carbon reduction; decarbonization; climate crisis; health crisis; net zero; zero emissions; healthcare emissions; sustainable healthcare; climate resilient healthcare; Paris Agreement; renewable energy; COP 26 Health Program; climate-smart solutions; climate change and health; climate change and healthcare. These terms were used to look for national level policies relating to climate change and health, health and hospital level policies or case studies across 73 countries identified from the nations that signed up to the COP26 Health Program and nations that have a Commonwealth Fund Country Profile on 1 September 2022 (Table 1) [14, 15]. The United Kingdom and the United States (US) were excluded from the review as existing work explores policies in these nations [11, 16]. Of the nations included in the review, 27 were high income countries, 12 were upper middle-income countries, 20 lower middle-income countries and 14 were low-income countries. Sixty-three of the 73 countries had signed up to the COP26 Health Program by September 2022. Insights from the desk-based review were supplemented with discussions with individuals working in climate change and health in England, Australia, Austria, Canada, France, Norway, and the Netherlands.

**Table 1:** List of countries included in the review and findings from review

Country	High / Low Income Country	National reference to Decarbonization	National reference to Healthcare Decarbonization	Examples of Decarbonization in hospital	Reference to adaptation / resilience	Reference to financial commitment to decarbonization
Argentina	UMIC	yes	no	no	yes	no
Australia*	HIC	yes	yes	yes	yes	yes
Bahamas	HIC	no	no	no	yes	no
Bahrain	HIC	yes	no	no	no	no
Belgium	HIC	yes	no	no	yes	no
Belize	UMIC	no	no	no	yes	no
Bhutan	LMIC	no	no	no	no	no
Brazil	UMIC	no	no	yes	no	no
Burkina Faso	LIC	no	no	yes	no	no
Canada*	HIC	yes	no	yes	yes	yes
Cape Verde	LMIC	no	no	no	no	no
Central African Republic	LIC	no	no	yes	yes	yes
Chile	HIC	no	no	yes	no	no
China	UMIC	yes	no	no	no	no
Colombia	UMIC	no	yes	yes	no	no
Costa Rica	UMIC	no	no	no	no	no
Democratic Republic of the Congo	LIC	no	no	yes	no	no
Denmark	HIC	yes	no	no	no	yes
Dominican Republic	UMIC	yes	no	no	no	yes
Egypt	LMIC	yes	no	yes	yes	no
Ethiopia	LIC	yes	no	yes	yes	no
Fiji	UMIC	yes	yes	no	yes	yes
France*	HIC	yes	no	yes	yes	no
Georgia	LMIC	yes	no	no	yes	no
Germany	HIC	yes	no	yes	no	no
Ghana	LMIC	no	no	no	no	yes
Guinea (Republic of)	LIC	no	no	no	no	no
India	LMIC	yes	no	no	yes	no
Israel	HIC	yes	no	no	no	no
Italy	HIC	no	no	no	no	no
Indonesia	UMIC	yes	no	no	no	no
Ireland	HIC	yes	no	no	no	no
Islamic Republic of Iran	LMIC	no	no	no	no	no
Ivory Coast	LMIC	no	no	no	no	no
Jamaica	UMIC	no	no	yes	yes	yes
Japan	HIC	yes	no	no	no	no
Jordan	UMIC	no	no	no	no	no
Kenya	LMIC	no	no	no	no	no
Lao PDR	LMIC	yes	no	no	no	no
Liberia	LIC	no	no	yes	no	no
Madagascar	LIC	yes	no	no	yes	yes
Malawi	LIC	no	no	no	no	no
Maldives	UMIC	yes	no	yes	yes	yes
Mauritania	LMIC	no	no	no	no	no
Morocco	LMIC	yes	no	no	no	no
Mozambique	LIC	no	no	no	no	no
Nepal	LMIC	yes	no	yes	yes	no
Netherlands*	HIC	yes	yes	yes	no	no
New Zealand	HIC	yes	yes	yes	no	yes
Nigeria	LMIC	yes	no	no	no	yes
Norway*	HIC	yes	yes	no	no	no
Occupied Territories of Palestine	LMIC	yes	no	no	no	no
Oman	HIC	yes	no	no	no	no
Pakistan	LMIC	yes	yes	yes	no	yes
Panama	HIC	no	no	no	no	yes
Peru	UMIC	no	no	no	no	no
Rwanda	LIC	yes	no	yes	yes	no
Sao Tome and Principe	LMIC	yes	no	no	no	no
Sierra Leone	LIC	yes	no	no	yes	no
Singapore	HIC	yes	yes	no	no	no
Spain	HIC	yes	yes	yes	no	no
Sri Lanka	LMIC	no	no	yes	no	no
Sweden	HIC	yes	yes	yes	no	no
United Kingdom	HIC	yes	yes	yes	yes	yes
United States of America	HIC	yes	yes	yes	no	no
Switzerland	HIC	yes	no	no	no	no
Taiwan	HIC	yes	yes	no	no	no
Tanzania	LMIC	yes	no	no	no	yes
Togo	LIC	no	no	no	no	no
Tunisia	LMIC	yes	no	no	no	no
Uganda	LIC	yes	no	no	no	no
United Arab Emirates	HIC	yes	no	no	no	no
Yemen	LIC	no	no	no	yes	no

^*^Gray literature review supplemented with discussions with national leaders working on health decarbonization

The abbreviations in the table refer to the following: HIC to High Income Country, UMIC to Upper Middle Income Country, LMIC to Lower-Middle Income Country and LIC to Low-Income Country

News articles, web pages, and other documents relating to national-level policy on health system decarbonization were included, the review also captured cross-cutting documents on health system decarbonization that are non-nation specific. Using this approach, 257 items were captured across 73 countries as well as a further 29 cross-cutting documents.

Literature and insights relating to county specific efforts for decarbonization were reviewed using a classification scheme adapted from Singapore’s decarbonization report, selected based on the level of detail and thoroughness of Singapore’s government’s report (see Table 2).

**Table 2:** Countries with national decarbonization policies in the literature review (*n* = 73 countries)

	Green economy	Green citizenry	Green living	Green energy	Green government
Australia*	yes	no	no	yes	yes
Austria*	no	no	no	no	no
Colombia	yes	no	no	yes	no
Fiji	yes	no	yes	yes	yes
France*	yes	yes	yes	yes	yes
Germany	yes	no	no	yes	no
Ireland	yes	yes	yes	yes	yes
Netherlands	yes	yes	yes	yes	yes
New Zealand*	yes	yes	yes	yes	no
Norway	yes	no	yes	yes	yes
Pakistan	yes	yes	yes	yes	yes
Singapore*	yes	yes	yes	yes	yes
Spain	yes	yes	no	yes	no
Sweden*	yes	no	yes	yes	no
Taiwan*	yes	no	yes	yes	no

Table summarizing the countries with national decarbonization strategies according to specific categories. Green energy refers to greener electricity and buildings (on low carbon and energy), LED lighting/solar energy, and cleaner vehicles with sustainable fuels. Green economy refers to planned or are planning to implement taxes on carbon and converge more to greener finance, production and job creations as well as developing sustainable tourism. Green living refers to naturalized infrastructures providing shade or promoting better air quality and water conservation through waste management and commutes. Green citizenry refers to measures, strengthening school curriculums with environmental classes, making younger populations environmentally aware at a younger age, but also keeping communities and stakeholders aware through partnerships (e.g., Maoris, NZ). And Green government support refers to any support for research and development, providing targets or enabling sustainable actions

This work has focused on a review of international policy and practice relating to health system decarbonization. With this focus, the review has not looked at evidence on the impacts climate change is having on human health and wellbeing, or the disproportionate and inequitable distribution of those impacts, though some of these insights have come through in the literature that has been reviewed.

## National Commitments to Reducing Healthcare Emissions

In November 2021 the UK Government, the World Health Organization (WHO), Healthcare Without Harm (HCWH) and the UNFCCC Climate Champions launched the COP26 Health Program [17]. The program, now known as the Alliance for Transformative Action on Climate and Health (ATACH), invited countries to sign up to two initial initiatives; to develop climate resilient health systems and low carbon sustainable health systems. At the time of the initial review, in September 2022, 60 nations had signed up to the program, 59 committed to developing climate-resilient health systems, 54 committed to sustainable, low-carbon health systems and 21 set a net zero commitment [18]. Since then, an additional 12 countries have made commitments. Of the nations that have committed to net zero health systems through ATACH, many appear to have made broader national commitments to reaching net zero across all sectors by 2050 or sooner. It therefore appears that it is this broader commitment that is driving the health systems’ targets, rather than a specific plan for action within the healthcare sector.

As much of the work on carbon emissions in healthcare is starting from scratch, some nations have started by focusing on understanding their baselines and building capacity and developing a roadmap or strategy for health system decarbonization. For example, in November 2021, Norway committed to reviewing the status of GHGs from the health sector and developing a roadmap to set the course for a sustainable low-emissions health sector by 2050 and net-zero operations in health trusts by 2045 [19]. Their goal is to establish the roadmap by 2023. This work is being revised and updated regularly through the annual social responsibility reporting for the hospital sector which includes updates on emissions, primarily for scopes 1 (direct emissions from sources an organization has control over) and 2 (indirect emissions from energy an organization purchases and uses) [20]. An approach for measuring scope 3 (indirect emissions from an organization’s wider supply chain) calculations is expected to be agreed in 2023. In February 2022, the Ministry of Health and Social Protection of the Republic of Colombia started a project with Healthcare Without Harm to estimate the climate footprint of the Colombian national health system at the facility level, with the project expected to report in 2023 [21]. In March 2023, the Australian Government launched a consultation on Australia’s first National Health and Climate Strategy which aims to set out a three-year plan for how the health system will manage the impacts of climate change and reduce its own emissions [22].

At the time this review was initiated (September 2022), the UK National Health Service (NHS) was the only nation to have formally published a clear roadmap for a net zero health system by a certain date (2045) [23]. However, since the review started a number of nations have initiated voluntary commitments to reduce emissions from healthcare, with some including emission reduction targets. In 2022, the Biden Administration invited health organizations to sign up to their Health Sector Climate Pledge. Over 100 health systems and industry organizations have joined in committing to reducing GHG by 50% by 2030, reaching net zero by 2050 and public reporting on progress [24]. Signatories also committed to appointing a designated executive-level lead for emissions reductions, undertaking an inventory of emissions from the supply chain and developing a climate resilience plan for services. In 2023, the Government of the Netherlands’ updated their Green Deal on Sustainable Healthcare to version 3.0, a voluntary commitment to develop more sustainable healthcare [25]. The new deal is more ambitious, has expanded its signatories to include the pharmaceutical industry and expanded to include five targets, which has been agreed to by over 200 hospitals and other care institutions [26]. These targets include focusing on health promotion of patients and staff; raising awareness of the impact of healthcare on the climate and the impact climate has on health; reducing emissions by 55% by 2030 and to net zero by 2050; reducing primary raw materials and maximizing circular healthcare; and reducing the environmental damage caused by medication. The Austrian Government has offered hospitals and care facilities consultancy support to review how to make their facilities more climate friendly. Over 300 institutions have signed up for this voluntary support, with reviews expected to be completed by 2024 providing recommendations for improvements across buildings, energy, food, waste, green space and other opportunities for health promotion.

Whilst many nations do not have specific policy on decarbonization of their health systems, they have policies and plans focused on decarbonization more generally. These are of interest, as broader action will help contribute to emissions reductions in healthcare. This research identified 15 countries with a clear national action plan for decarbonization published by their government. In line with the Paris Agreement, all countries identified have committed to limiting global average temperature rise to no more than 1.5 °C and reducing their greenhouse gas emissions by 55% by 2030. Emission reduction policies target energy, transport, buildings, water resources and waste management amongst others. Table 2 reports country level efforts for decarbonization using a classification scheme adapted from Singapore’s decarbonization report.

Despite not having a national healthcare decarbonization plan, many health systems and hospitals are still taking action to reduce their carbon emissions. There are many examples of this across the countries reviewed here (Table 3).

**Table 3:** Case studies on reducing emissions in hospitals

Country	Provider	Description
Canada	Centre Hospitalier Universitaire de Montréal (CHUM)	• Installed new anesthetic stations to minimize gas leaks, as well as modules that capture gasses before they escape through the hospital’s chimney [55]. With these changes, the anesthesia department expects to reduce its greenhouse gas emissions from 3,500 tons in 2017 to 150 tons by 2023 • The bill is already lighter than it was previously: anesthetic gas costs for the CHUM dropped from $532,000 in 2017 to $106,000 in 2022
Chile	Valdivia Public Health Network	• Shut down their incinerator • Hired environmental managers for each facility, creating an environmental leadership committee • Staff was trained to use the Health Care Without Harm carbon emission footprint tool to calculate their hospital’s emissions. This led to them finding out that in seven out of the eight facilities that calculated their carbon footprint, scope 1 emissions (those produced on site) represented more than half of total emissions, mainly caused by boilers, cooling and anesthetic gasses. Energy audits have since then been carried out to monitor and optimize energy efficiency (i.e. switching from fuel boilers to gas) • Built onsite orchards to educate staff, patients and the broader community on sustainable agricultural practices and providing nutritious, low-carbon and chemical-free food for the facilities [56]
France	Centre Hospitalier de Niort	• Current waste policies encourage the sorting and recycling of waste (cardboard, toxic waste from laboratories, computer waste, batteries etc.) to minimize the quantities of materials going to landfill or incineration [57] • Awareness campaigns and staff education to encourage these recycling behaviors have been implemented
New Zealand	Hawke’s Bay District Health Board – Go Well Initiative	• In 2014/15 the facilities management team developed a Go Well Travel Plan to (1) improve access for low-income families and whānau, (2) promote exercise, (3) reduce the carbon footprint of the hospital and (4) increase the availability of car parks [58] • Introduced measures to encourage visitors, staff and patients to use public transport (free bus fares for patients) and car pools • Since the implementation of the Go Well Travel plan, use of buses has increased by 100% among patients and 4% by staff, the proportion of staff driving cars has reduced by 10% and the proportion of cycling to work has increased by 3%
New Zealand	Forté Hospital	• First hospital in Australasia to achieve a four-star green certification through the New Zealand Green Building Council [59] • Initiatives they have implemented include: (1) Better segregation of waste resulting in a 47% reduction in the amount of medical hazardous waste requiring emission-producing heat treatment (2016 – 2021); (2) Reducing waste to landfill by 1.6 tons from 2019 to 2020; (3) Reducing electricity usage by 12% from 2019 to 2021; (4) Recycling staff uniforms into bags for patient belongings or donating them to Pacific Island hospitals, thanks to a partnership with Rotary Worldwide; (5) Installation of a new VIE liquid oxygen tank resulting in an approximately 99% reduction in CO2 emissions from truck deliveries; (6) Removed single-use food packaging for patient meals, saving around 1,200 single-use containers going to landfill each year and (7) Replaced medicine trays with compostable alternatives • Future sustainability goals include to further reduce its carbon dioxide equivalent emissions by 10% over the next five years • Carbon offsetting projects involving planting trees at Spray Point Station in South Marlborough, at Puhi Peaks Station and Nature Reserve in Kaikōura and at the Hinewai Reserve, Banks Peninsula
Norway	NA	• Used drone technology in 2021 to transport biological samples between cities as far as 120 km apart. [20] • Drones can potentially be faster, competitive on price and emit 95% less CO2 than cars and be available at any time
Singapore	Changi General Hospital	• Deployed solar panels back in 2017, which has helped generate about 50MWh of energy a year [60] • Current solar energy accounts for around 1% of the hospital’s annual energy use, covering non-critical systems such as roof lighting and fans. The hospital aims to ramp up to 5% solar generated energy through expansion to new rooftop spaces, which could see carbon emissions reduced by up to 4,000 tons over 25 years • Introduced a heat recovery system for hot water since 2002, using waste heat from the air-conditioning system to heat water for showers and sterilization processes. Replacing water heated by the hospital’s gas-fired heaters has saved $300,000 a year

## Actions and Enablers to Reduce Emissions from Healthcare

As part of the review a number of documents were identified that cover issues related to climate change and health but were not country specific. Twenty-nine documents were identified and reviewed in detail to draw out the key themes, guidance and policy recommendations related to climate change and health. Eighteen of the documents focused on mitigation in healthcare, four focused on climate smart or sustainable healthcare, two on adaptation and the remaining focused on healthcare carbon footprints; resilience, mitigation, and adaptation in healthcare; ethics of medicine disposal; non-healthcare specific impacts of climate change and non-health specific mitigation and adaptation strategies.

The reviewed documents were published between 2005 and 2023, with the majority of (16 out of 29) published in 2021 (8) and 2022 (8). Fifteen of the documents were reports that included policy recommendations or guidance, seven were journal articles, two documents provided frameworks for healthcare system resilience or sustainability, two were commentaries, two included analyses relating to carbon footprint and two were blogs.

Table 4 sets out the areas of opportunity that were most commonly referenced as ways to drive decarbonization in health systems. The area most frequently referenced was energy, specifically increasing use of clean, renewable energy and increasing energy efficiency, followed by supply chain, including pharmaceuticals. Other frequently referenced areas include healthcare buildings, infrastructure and transport. References to transforming clinical care and increasing the focus on prevention were also common, but references tended to focus on the need for more innovation to reduce emissions, rather than providing example initiatives that could be implemented.

**Table 4:** Areas of opportunity to drive decarbonization in health systems taken from cross-cutting documents

Area	References (*n* = 29)	Opportunities for decarbonization
Energy	18	- Increase use of clean, renewable energy and reduce reliance on fossil fuels - Increase energy efficiency, e.g. LED lighting, energy audits
Supply chain (inc. pharma)	18	- Low carbon procurement policies and incentives - Regulation of new products to include consideration of carbon footprint - Minimize use of high GHG-emitting anesthetic gases - Life cycle analysis across the supply chain - Innovation with a focus on low-carbon pharma - Innovation in product design and packaging
Clinical care transformation and prevention	15	- Design new models of care to make use of new technologies and coordinated care - Optimize care pathways for efficiency - Reduce over treatment and unnecessary tests - Increase personalized and targeted care - Focus on more preventative / anticipatory care - Understand different carbon utilization across different procedures - Consider options for reuse across a circular economy
Buildings and facilities	15	- Adopt green features in existing buildings - Update building standards / requirements to be ‘green’ - Update heating and cooling systems - Improve insultation - Sustainable sourcing - Low carbon design and construction - Consider opportunities to increase efficiency in use of estate - Greening of operating theatres
Travel & transport	12	- Sustainable transport planning - Minimize need for high emitting transport - Focus on increasing active transport, which also has health co-benefits
Digital infrastructure	11	- Develop digital infrastructure and processes to help reduce emissions and increase efficiency in care delivery - Use of telemedicine
Water, waste and sanitation	11	- Focus on sustainable water management - Improve waste management - Use of safe and environmentally friendly chemicals
Food	9	- Promote sustainable, healthy diets - Source local, sustainably grown foods - Minimize food waste - Reduce meat in favor of plant-based foods - Encourage regenerative agriculture
Workforce	8	- Embed sustainability in organization’s culture and values - Employ sustainability lead - Staff training - Workforce as a driver for sustainability action, from current staff and as recruitment incentive

The high number of references to reducing emissions from energy within the reviewed documents reflects the scale of the opportunity in the sector. In some countries, legislation and regulation is already incentivizing a shift to more renewable energy across the whole economy which will ultimately benefit healthcare [12, 27]. However, given energy requirements within healthcare, it could also help drive a faster shift away from fossil fuels by demanding more clean, renewable energy sources. Where they have the capacity to do so, hospitals can go further than this and use their own resources to generate energy, potentially affording additional income. One of the prime examples of this in both high- and low-income countries is the introduction of solar panels to provide energy to help run hospitals, and increase energy security [28, 29].

The supply chain is the greatest source of emissions across the healthcare sector (see Fig. 1); the need to reduce emissions from the supply chain and the pharmaceuticals sectors was referenced in the majority of documents that were reviewed. Whilst there were some specific references to opportunities to reduce emissions, such as reducing high-GHG emitting anesthetic gasses, how the sector will meet the challenge of reducing emissions from the supply chain seems less clear. A number of the documents referenced the need for more regulation, legislation or incentives to enable more low-carbon procurement. However, there were few specifics on how that could be practically delivered. Some health systems have introduced ‘green’ or ‘low carbon’ procurement, this often means increasing transparency in reporting on emissions from suppliers and requiring providers to have plans to reduce their emissions [30•, 31•]. Recognizing new solutions will be needed, innovation in the development of new sustainable products and packaging was referenced by several documents [32, 33]. This reinforces the view that more work is required to develop a truly low-carbon supply chain [34].

After energy and supply chain, the area referenced most frequently was the need for clinical transformation and a shift to more prevention to reduce emissions in healthcare. Reducing over-treatment, shifting to more targeted, personalized, and preventative care, and investing in public health infrastructure can reduce future demand for healthcare services and therefore remove associated emissions entirely [35, 36•]. In addition to this, there are benefits to maximizing technology to support more efficient care pathways and reduce emissions associated with travel [37]. Some documents also highlighted that understanding carbon utilization of different healthcare procedures could help clinicians and staff identify lower emission procedures and make positive daily changes [38]. Specific clinical areas that were referenced as being high carbon were anesthetics, operating rooms and endoscopy [39].

Many of the other areas of decarbonization that were frequently referenced in the cross-cutting documents are not unique to healthcare. Improving the design and construction of buildings [40, 41], reducing emissions from travel and transport, improving waste and water management and food sourcing are all areas of opportunity that apply across a wide range of sectors [42–44]. Many of the suggested initiatives will also have a broader health co-benefit. Increasing active travel can provide health benefits for individuals, improving waste disposal can reduce environmental toxins and good building design with access to more green space can aid recovery and wellbeing. In drawing on best practice from other sectors, healthcare can not only reduce their own carbon footprint, but also support improved health and wellbeing.

In addition to discussing what health systems can do to reduce their emissions, many of the reviewed documents suggested enablers that could help support implementation. Several of the documents set out approaches to developing a plan for reducing emissions, the key steps for which include understanding baseline emissions; setting clear targets to work towards developing a plan that includes specific direct and indirect initiatives and establishing reporting on agreed metrics to track progress against targets.

A number of enablers that can help facilitate delivery of the plans were also discussed in a number of documents. The most commonly reference enablers were the need for leadership support for the agenda and funding to support action. Other areas that were highlighted as priorities were collecting and reporting on agreed metrics to track progress and regulation to drive change and prevent ‘greenwashing’, ensuring that the benefits of action to protect the environment are not misrepresented or overplayed [45]. The importance of establishing effective peer partnerships and collaboration to help drive change was also referenced as a mechanism to increase scale of influence, the value of collaborations to share learning, create accountability and bring together cross-functional expertise [46]. Supporting the workforce through effective training can help staff take action to reduce emissions, but also support the clinical workforce consider how to include climate change considerations in their treatment pathways. Finally, innovation and new solutions was considered to be an important enabler by some publications. Research and development will be needed to close the gaps in reducing emissions across the healthcare sector. These innovations will need to be evidence-based and grounded in science, but also support the necessary pace of change required for healthcare to make its contribution to reducing global emissions.

## Global Variations in Focus on Mitigation and Adaptation 

Not all nations focused on mitigation; some have a greater focus on adaptation to the impacts of climate change. Many of those who had a greater focus on adaptation, rather than mitigation, were low- and middle-income countries (LMIC). LMICs are often at greater risk of impact from climate change but less well prepared to respond to those risks, despite their emissions often being relatively low. For example, Bangladesh emits just 0.5% of the world’s emissions, but has been significantly impacted by climate change through increased flooding and cyclones in recent years [47]. In contrast to this, higher income nations (HIC) are often higher emitters but have better planning and capacity to respond to climate change-related impacts and emergencies.

One of the key approaches nations are taking to respond to climate change is to develop and publish a National Adaptation Plans, which highlights their vulnerabilities to climate change and plans to adapt, including plans to strengthen and protect health and healthcare services [48]. These plans often link with requirements to meet the Sustainable Development Goals and National Determined Contributions (NDCs), with many drawing on international resources and frameworks, such as the WHO guidance on climate resilient and environmentally sustainable healthcare facilities [49]. To deliver on these plans, many LMICs will require financial support and investment from initiatives such as the World Bank, the Green Climate Fund, or International Development funding such as UK Aid or USAID [50]. These investments often require low-carbon solutions that help support expansion of healthcare access and low-carbon solutions, often driving innovation and a stronger infrastructure for the future.

Some nations have also developed national climate change policies that highlight the inequity of climate change, particularly gender gaps in facing climate change [51–53]. To address these issues, Fiji, Pakistan, and Singapore are among those that have described measures to include gender and climate change into school curricula and management and including women and the youth to play a role in climate action, helping to reduce inequalities.

International conferences, such as COP and WEF, continue to highlight the interconnectivity between climate change and health, with many discussing how to support health systems in both mitigation and adaptation. This will hopefully keep nations focused on prioritizing action on both mitigation and adaptation within their health systems, and continue to channel investment to support delivery from governments and global foundations.

## Conclusions

This review demonstrates that there is recognition of the role that health systems have to play in reducing emissions to mitigate climate change and ‘do no harm’, which is the first duty of all physicians within the Hippocratic Oath. Global health systems are starting to recognize the role they have to play, with some starting to respond and act. Much of this initial action is voluntary, from those that are willing and able to take positive action to reduce their emissions. This is a positive first step, but it will be important that voluntary national and international commitments are not notional and lead to true action, not greenwashing or misleading statements on progress.

Looking across the breadth of reports that have been published in this space, there are some clear actions that all health systems can take to reduce their emissions. Many of these actions are not unique to healthcare and some are being supported by broader incentives to reduce emissions from energy, buildings, transport and food waste. There are a number of frameworks, like the one developed by Global Green and Healthy Hospitals [54], that aim to describe both the issue and the action opportunities for hospitals.

These frameworks are helping in setting out the known actions that health systems can take to reduce emissions. However, they will not be sufficient for those looking to reach net zero, or become carbon neutral health systems. Innovation across clinical care, supply chain and research and development will be required to fully close the gap to carbon neutrality. Many of the reports and recommendations have highlighted the importance of shifting to better prevention and more community-based care in lower-carbon settings. However, these are issues that the health system has been grappling with for years, or decades, and has not yet achieved. It is not yet clear if the climate crisis will be sufficient to finally shift the balance.

To reach net zero across the whole healthcare supply chain is likely to require transformation, particularly energy transformation, globally. Fossil fuels are still heavily used in some countries further up the supply chain and in R&D. Transformation in those nations may require international action and investment.

The strengths of this review include the breadth of nations reviewed and the diversity of literature considered. Insights from interviews with experts across a number of nations has further strengthened the findings. There were three main limitations to this review. There were limited open publication of policy documents at the time of the review (September–October 2022). The review was limited to publications in English and French. Lastly, the search was completed from within the US, which may have impacted responses from the web-based search engine.

Whilst this review did not explicitly look at the impacts of climate change on human health and wellbeing, or the inequity of those impacts, this has come through in some of the literature. Many of the documents reviewed recognize the distribution of risk from climate-related events don’t fall equally, with greater risks often facing more vulnerable populations. Such inequities are not just a global issue, the impacts of climate change are likely to impact US populations differently as well. More research is needed to understand how climate change is confounding existing inequities, whether more targeted efforts to reduce emissions in areas with higher risk populations could help address some of the existing health inequities that exist across the US and how the inequitable impact of climate change requires targeted adaptation to support vulnerable populations from the increasing scale and frequency of climate impacts.
